# Peroneal Nerve Palsy With Foot Drop: A Rare Manifestation of Oxaliplatin Toxicity

**DOI:** 10.7759/cureus.95932

**Published:** 2025-11-02

**Authors:** Marisa C Couto, Maria J Ribeiro, Miguel Barbosa, Nuno T Tavares

**Affiliations:** 1 Medical Oncology, Unidade Local de Saúde de São João, Porto, PRT

**Keywords:** foot drop, neurotoxicity, oncology, oxaliplatin, peroneal nerve palsy

## Abstract

Oxaliplatin is a platinum-based chemotherapy drug that is distinguished from others in its class by its neurotoxicity. This toxicity most commonly presents as a peripheral neuropathy with sensory symptoms, and less frequently as motor or autonomic disorders. Peroneal nerve palsy is a mononeuropathy presenting with foot drop and may result from multiple causes. Its occurrence in association with chemotherapy, particularly oxaliplatin, is rare.

We report two cases of peroneal nerve palsy associated with oxaliplatin treatment. Case 1 involves a 73-year-old female with stage IV pancreatic adenocarcinoma who developed a left foot drop after four cycles of modified FOLFIRINOX (oxaliplatin 85 mg/m²). Neurological examination revealed distal weakness (3/5) without additional deficits. Electromyography confirmed axonal and demyelinating injury of the left common peroneal nerve (CPN). Other causes were ruled out. Following oxaliplatin discontinuation, the deficit completely resolved. Case 2 involves a 62-year-old male diagnosed with adenocarcinoma of the gastroesophageal junction who underwent neoadjuvant chemoradiotherapy with carboplatin (AUC 2) plus paclitaxel (50 mg/m²) and presented with metastatic disease five months later. After four cycles of FOLFOX (oxaliplatin 85 mg/m²), he developed a painless right foot drop with isolated motor deficit (0/5). Laboratory tests, brain CT, and spine MRI excluded alternative causes. Disease progression precluded further evaluation, and the patient died before the clinical course of the palsy could be assessed.

These two cases highlight the potential of oxaliplatin-induced neurotoxicity to manifest as an isolated peroneal nerve palsy with foot drop. Given that only six cases of this condition have been described in the literature so far, further case reports are essential to better characterize risk factors, clinical course, and outcomes of this rare complication.

## Introduction

Oxaliplatin is a third-generation platinum-based chemotherapy drug used in various cancers of the gastrointestinal tract. Its main side effects include peripheral neuropathy, myelosuppression, and digestive disturbances such as nausea and diarrhea [1]. Oxaliplatin’s neurotoxicity is linked to its direct action on neuronal ion channels - particularly sodium channels - increasing the duration of the nerve action potential (NAP) and reducing the amplitude of the trough following the NAP peak. These changes can persist even after drug removal [2]. Neurotoxicity of oxaliplatin can cause sensory, motor, and autonomic symptoms. In clinical practice, the majority of patients present with peripheral neuropathy characterized by distal sensory disorder, changes in proprioception, and deep tendon reflex suppression [1]. Motor and autonomic symptoms are less frequent.

The common peroneal nerve (CPN) palsy is the most frequently encountered mononeuropathy of the lower limb. This nerve is a branch from the sciatic nerve and is responsible for dorsiflexion and eversion, as well as sensory innervation of the upper lateral third of the foot. The palsy of the peroneal nerve can manifest as a unilateral foot drop [3,4]. Foot drop results from weakness of the dorsiflexor muscles of the ankle and is most commonly caused by an injury in the lower motor neuron. Examples of etiologies affecting this neuron include compression of the CPN as it courses around the fibular head, sciatic mononeuropathy, and lumbosacral plexopathy [5]. As for central etiologies, it can be caused by a vascular event or space-occupying lesion involving the places where fibers are highly condensed along the upper motor neuron [6]. Other rare causes of foot drop include weight loss, multiple sclerosis, paraneoplastic syndromes, and chemotherapy [3-5]. A quick, structured evaluation for both peripheral and central causes can save time and guide the right workup.

Isolated motor impairment from oxaliplatin, such as muscle weakness or palsy, is rarely described, and its underlying mechanism remains poorly understood. In fact, to our knowledge, only six such cases have been documented in the literature to date [4,7]. We report two additional cases of isolated peroneal nerve palsy presenting as foot drop following chemotherapy with several cycles of oxaliplatin.

## Case presentation

Case 1

The patient was a 73-year-old Caucasian female with an Eastern Cooperative Oncology Group (ECOG) performance status (PS) of 0. Her relevant medical history included dyslipidemia and well-controlled type 2 diabetes mellitus, without evidence of target organ damage, such as peripheral neuropathy: treated with atorvastatin 40 mg plus ezetimibe 10 mg and dapagliflozin 10 mg, respectively. She had a smoking history of 58 pack-years and an alcohol consumption of approximately 50 g/day. There was no history of vitamin B12 deficiency, hypothyroidism, or other known risk factors for neuropathy. She had no known drug allergies. Her family history was notable for a mother who died at 66 from breast cancer and a maternal grandmother diagnosed with gastric cancer.

The patient was diagnosed with pancreatic ductal adenocarcinoma in January 2025 after presenting to the emergency department with upper abdominal pain. A CT scan revealed a hypodense nodule in the pancreatic tail measuring 18 × 16 mm, along with multiple hypodense liver lesions with ring enhancement, suggestive of secondary deposits. MRI confirmed the presence of multiple retroperitoneal and mesenteric lymph node metastases, hepatic metastases, left adrenal involvement, and bone metastases. A biopsy of one hepatic metastasis confirmed the diagnosis. The case was reviewed at a multidisciplinary tumor board, and systemic palliative therapy was advised.

The patient was initiated on treatment with a modified FOLFIRINOX regimen (oxaliplatin 85 mg/m², irinotecan 180 mg/m², leucovorin 50 mg, fluorouracil 400 mg/m² bolus, and 2400 mg/m² continuous infusion over 46 hours) every 14 days. She received the first cycle on February 14, 2025, and after four cycles (cumulative oxaliplatin dose of 340 mg/m^2^), developed left foot drop, with distal strength graded 3/5, without other neurological abnormalities on examination.

**Figure 1:**
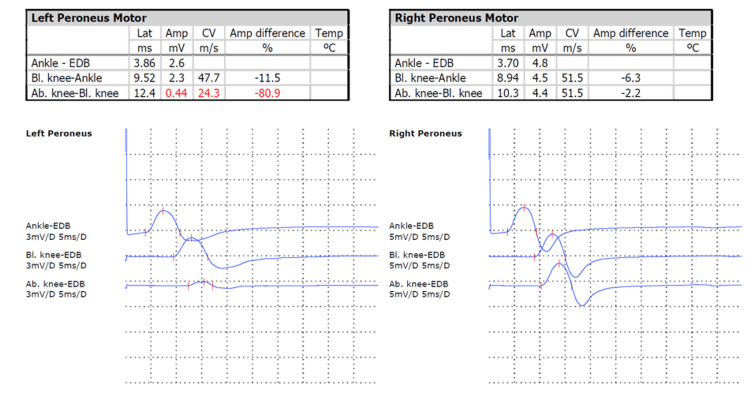
Electromyography revealing demyelination of the left common peroneal nerve (Case 1) Motor nerve conduction studies of the peroneal nerve demonstrate prolonged distal latency and markedly reduced conduction velocity on the left side (24.3 m/s) compared with the right (51.5 m/s), consistent with a demyelinating pattern. There is also a significant amplitude reduction between stimulation sites above and below the knee on the left (-80.9%), suggesting partial conduction block. The right peroneal nerve shows normal conduction parameters Lat: latency (A-axis); Amp: amplitude (Y-axis); CV: conduction velocity; EDB: extensor digitorum brevis muscle; Bl.: below; Ab.: above

A whole-body CT scan demonstrated partial response of the oncologic disease and excluded lumbosacral spinal involvement by bone metastases with spinal cord or nerve root invasion. Brain CT scan ruled out intracranial metastases. Electromyography confirmed axonal and demyelinating injury of the left CPN, with evidence of mild acute denervation in muscles innervated by the superficial and deep peroneal nerves, and markedly reduced motor unit recruitment in muscles of the deep peroneal nerve in the left leg and foot (Figure 1). The remaining peripheral motor and sensory nerve conduction studies in the lower limbs were normal, with no electrophysiological evidence of polyneuropathy. Blood tests showed no significant changes.

Following the discontinuation of oxaliplatin, there was a complete and gradual resolution of symptoms over the following six weeks. The patient subsequently continued chemotherapy with the FOLFIRI regimen (irinotecan 180 mg/m², leucovorin 50 mg, fluorouracil 400 mg/m² bolus, and 2400 mg/m² continuous infusion over 46 hours).

Case 2

The patient was a 62-year-old caucasian male with an ECOG PS of 1. His relevant medical history included hypertension, first-degree atrioventricular block, and untreated type 2 diabetes mellitus with an HbA1c of 7.5%, without known target organ damage. Surgical history was notable for appendectomy, left inguinal hernioplasty, and testicular surgery for hydrocele. He was a former smoker with a 40 pack-year history and reported no alcohol consumption. There was no history of vitamin B12 deficiency, hypothyroidism, or other known risk factors for neuropathy. He had no known drug allergies, and his family history was negative for cancer or hereditary diseases.

The patient was diagnosed in June 2024 with a cT2N+M0 adenocarcinoma of the gastroesophageal junction, following an upper endoscopy performed for progressive dysphagia. The case was discussed at a multidisciplinary tumor board, which recommended neoadjuvant chemoradiotherapy. He completed five cycles of carboplatin (AUC 2) plus paclitaxel (50 mg/m²) weekly, concurrently with radiotherapy (45 Gy in 25 fractions) from July 15 to August 20, 2024.

A restaging CT scan after neoadjuvant therapy demonstrated a partial response, and on October 15, 2024, he underwent a minimally invasive subtotal esophagectomy (Ivor Lewis procedure). Histology revealed an adenocarcinoma of the gastroesophageal junction with tumor regression grade 3, showing more than 50% of residual disease in the tumor bed. One of seven excised lymph nodes was positive for metastatic disease, corresponding to ypT3N1R0. Although the pathological report did not specify the “M” category, radiological assessment at that time was consistent with M0 disease. Molecular testing showed no microsatellite instability, HER2 1+, and PD-L1 CPS 14. After the resolution of postoperative wound infection, the patient was initiated on adjuvant therapy with nivolumab 480 mg every 28 days in December 2024.

A follow-up thoracoabdominal CT scan in January 2025 demonstrated new-onset multiple liver metastases and retroperitoneal lymphadenopathy. The patient was proposed for systemic palliative chemotherapy with the FOLFOX regimen (oxaliplatin 85 mg/m², leucovorin 50 mg, fluorouracil 400 mg/m² bolus, and 2400 mg/m² continuous infusion over 46 hours) every 14 days. He began treatment on February 28, 2025, and after the fourth cycle (cumulative oxaliplatin dose of 340mg/m^2^), developed painless right foot drop, without other neurological deficits.

**Figure 2:**
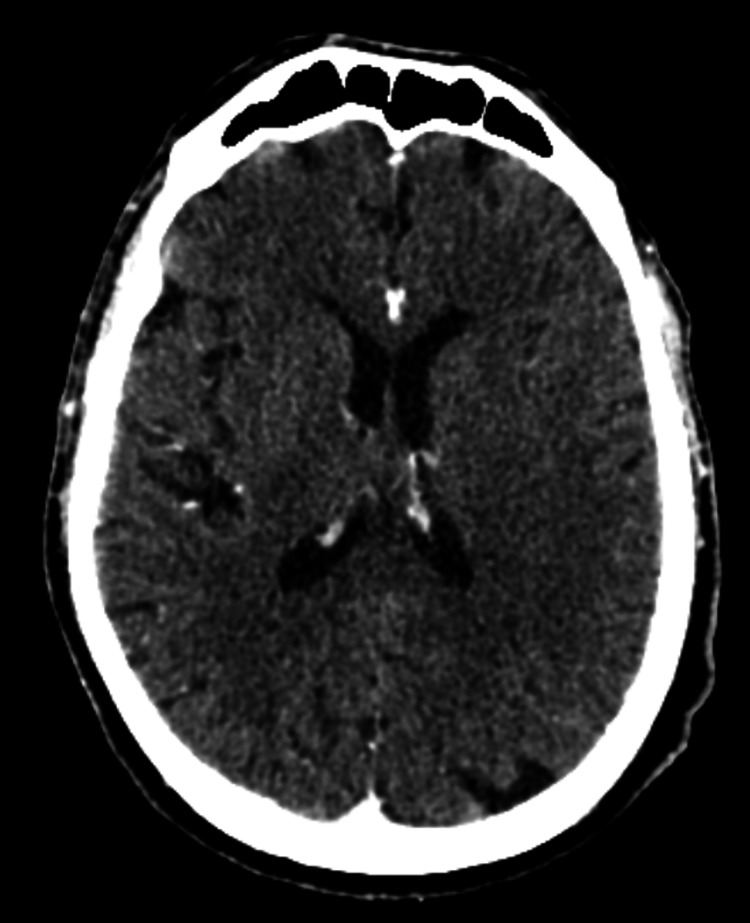
Brain CT scan showing no metastatic disease or acute findings (Case 2) CT: computed tomography

**Figure 3:**
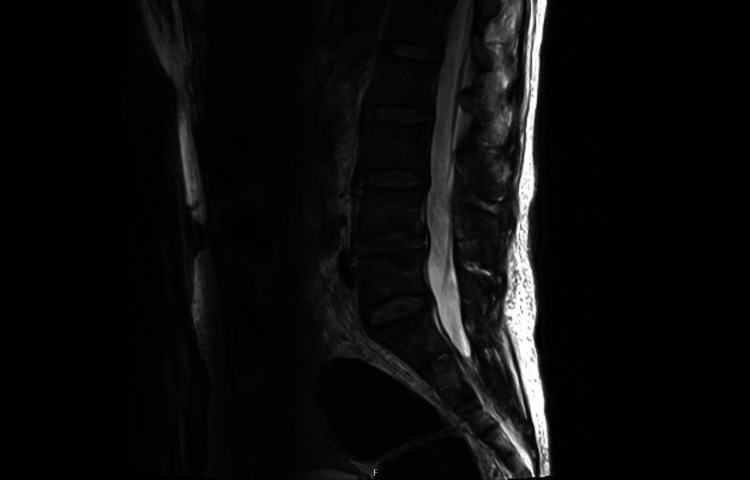
Lumbosacral spine MRI showing no secondary infiltration or radicular involvement (Case 2) MRI: magnetic resonance imaging

Neurological evaluation revealed an isolated motor deficit of the right foot, with muscle strength 0/5 and a positive plantar reflex (Babinski sign). Due to suspicion of upper motor neuron lesion, the patient underwent brain CT (Figure 2), which showed no evidence of metastatic disease or acute/subacute vascular events; and lumbosacral spine MRI (Figure 3), which revealed no suspicious secondary infiltration or spondylodiscopathy with radicular involvement. The blood test did not reveal any new or relevant changes. A whole-body CT demonstrated extensive retroperitoneal adenopathic conglomerates, without nerve root invasion. A trial of corticosteroid therapy was attempted for a suspected late immune-related neurological adverse event secondary to nivolumab, without improvement.

The patient’s clinical condition worsened, requiring hospitalization due to progression of oncologic disease, with hepatic dysfunction and anorexia-cachexia syndrome. Electromyography was not performed, and care was shifted to best supportive management due to his poor functional status and dependency. The patient died on May 17, 2025.

## Discussion

The two cases reported here illustrate the occurrence of isolated CPN monoparesis, likely associated with oxaliplatin therapy. Clinically, the presence of foot drop should prompt consideration of chemotherapy-induced neurotoxicity as a potential differential diagnosis once other causes related to lower or upper motor neurons have been excluded. Foot drop as a manifestation of chemotherapy-induced toxicity is not yet widely recognized, and clinicians across specialties should remain vigilant for this unusual neurotoxicity. In fact, the only consistent finding across all cases described in the literature and our two cases is as follows: a cause-and-effect relationship following several cycles of oxaliplatin, and a complete and spontaneous resolution of neurological deficits after discontinuation of the drug (Table 1).

**Table 1:** Foot drop cases during oxaliplatin treatment: cumulative dose at onset and time to resolution F: female; M: male; Adeno: adenocarcinoma; RT: radiotherapy; POD: progression of disease

Patient	Age/sex	Diagnosis and stage	Chemotherapy regimen	No. of cycles before onset	Cumulative onset dose of oxaliplatin	Time to resolution
A [4]	49/F	Rectal Adeno T3N1cM0	Neoadjuvant capecitabine 1000 mg/m² + RT, adjuvant capecitabine 1000 mg/m² + oxaliplatin 130 mg/m² every 21-day cycle	4	520 mg/m²	4 months
B [4]	85/M	Colon Adeno TxNxM1	mFOLFOX6 (oxaliplatin 85 mg/m^2^ + leucovorin 400 mg/m^2^ + fluorouracil 400 mg/m^2^ + fluorouracil 2400 mg/m^2^) every 14-day cycle	5	425 mg/m²	6 months
C [4]	54/M	Gastric – signet ring TxNxM1	FOLFOX for 6 cycles with POD, switched to ramucirumab 8 mg/kg + paclitaxel 80 mg/m^2^ (days 1, 8, and 15 of 28-day cycle)	6	510 mg/m²	4 months
D [4]	75/M	Gastric Adeno T4bN0M1	Perioperative FLOT (docetaxel 50 mg/m² + oxaliplatin 85 mg/m² + fluorouracil 2600 mg/m² every 14-day cycle) followed by capecitabine 1000 mg/m² + RT	8	685 mg/m²	2 months
E [7]	59/F	Colon Adeno T3N1M1	Palliative FOLFOX and panitumumab every 14-day cycle	3	255 mg/m²	1 month
F [7]	54/M	Colon Adeno T3bN2aM0	Adjuvant FOLFOX every 14-day cycle	3	255 mg/m²	2 weeks
Case 1	73/F	Pancreatic Adeno TxNxM1	Palliative mFOLFIRINOX every 14-day cycle	4	340 mg/m²	6 weeks
Case 2	62/M	Gastroesophageal junction Adeno T3N1M1	Palliative FOLFOX every 14-day cycle	4	340 mg/m²	Died 1 month after the onset

Effectively, the number of oxaliplatin cycles preceding the onset of foot drop appears to be similar among the cases described, with a median of 4.5 cycles (minimum of three and maximum of eight), consistent with the treatment exposure in the two patients presented here. The cumulative dose ranged from 255 to 685 mg/m^2^. The patients who developed neuropathy at lower cumulative doses had no reported risk factors or pre-existing neuropathy.

Clinicians might consider closer neurological monitoring between the third and eighth cycles of oxaliplatin, when the risk of neurotoxicity seems to peak. Electromyography should be performed to confirm the diagnosis, and other potential causes should be ruled out; in case 2, the inability to perform this examination due to the patient’s deterioration represents a limitation of this report. Regarding recovery, all reported cases achieved full resolution of motor deficit after oxaliplatin withdrawal, with recovery times ranging from two weeks to six months. To date, there have been no reports of oxaliplatin-related foot drop with irreversible or chronic motor deficits [4,7].

Furthermore, it is also important to note the potential contribution of other anticancer agents, such as paclitaxel, capecitabine, and vincristine, to the development of peroneal nerve palsy. In fact, among the six cases described in the literature, three involved concomitant or sequential administration of taxanes or capecitabine with oxaliplatin. Although it remains unclear whether these combinations exacerbate this neurological toxicity, the possibility of increased risk in patients treated with multiple anticancer drugs should be considered [4]. Moreover, given that there are currently multiple combinations of antineoplastic drugs from various classes, such as chemotherapy regimens combined with immunotherapy, it is necessary to rule out an immune-mediated etiology for peroneal nerve palsy [8]. Another hypothesis raised in the literature is that oncologic patients may have an inherently increased risk of developing peroneal nerve palsy, influenced not only by chemotherapy but also by additional metabolic and mechanical factors (reported in up to 16% of cases) [9]. These include weight loss (60% of patients), leg crossing (35%), cutaneous vasculitis (5%), and local metastatic lesions (3%) [10].

Regarding the treatment of oxaliplatin-induced foot drop, there is currently insufficient evidence to support the use of pharmacological agents typically employed in oxaliplatin-related peripheral neuropathy, such as duloxetine. Some authors recommend physical rehabilitation under the supervision of a physical therapist - including passive and active range-of-motion exercises and muscle strengthening - as well as the use of an ankle-foot orthosis to provide stability and prevent ambulation-related injuries until recovery of active movement. However, discontinuation of the offending agent, in this case oxaliplatin, remains the only intervention consistently associated with symptom resolution [4].

Concerning risk factors, it should be taken into account that certain comorbidities may increase the likelihood of developing peripheral neuropathy, such as diabetes, particularly in cases of poor glycemic control, as observed in case 2. Nevertheless, as no preventive strategies have yet been established for chemotherapy-induced neuropathy, oxaliplatin-related CPN palsy currently lacks both evidence-based prevention and clear identification of patient-specific risk factors that may predispose certain individuals to this complication [4]. Hence, awareness of this potential adverse event is crucial, as it is infrequently reported and remains poorly understood from a pathophysiological perspective. Maintaining vigilance and sharing such rare clinical experiences can improve patient care and facilitate early recognition of subtle motor changes suggestive of foot drop.

## Conclusions

The two cases presented here add to the limited reports in the literature of peroneal nerve palsy as a potential complication of oxaliplatin therapy in cancer patients. The temporal relationship between oxaliplatin exposure and the onset of peroneal nerve palsy, along with the improvement observed after drug discontinuation, supports a likely causal association. Although uncommon, this form of neurotoxicity should be recognized by healthcare professionals as a differential diagnosis in patients presenting with foot drop. Early identification in clinical practice is essential to enable discontinuation of the drug and facilitate reversal of the motor deficit, which, if unaddressed, significantly impairs patients’ ability to perform activities of daily living.
